# LKB1 inactivation leads to centromere defects and genome instability via p53-dependent upregulation of survivin

**DOI:** 10.18632/aging.103473

**Published:** 2020-07-16

**Authors:** Li-Yan Jin, Kui Zhao, Long-Jiang Xu, Rui-Xun Zhao, Kaitlin D. Werle, Yong Wang, Xiao-Long Liu, Qiu Chen, Zhuo-Jun Wu, Ke Zhang, Ying Zhao, Guo-Qin Jiang, Feng-Mei Cui, Zhi-Xiang Xu

**Affiliations:** 1 State Key Laboratory of Radiation Medicine and Protection, School of Radiation Medicine and Protection, Soochow University, Suzhou 215123, China; 2 School of Life Sciences, Henan University, Kaifeng, Henan Province 475004, China; 3 Department of General Surgery, The Second Affiliated Hospital, Soochow University, Suzhou 215004, China; 4 Department of Pathology, The Second Affiliated Hospital, Soochow University, Suzhou 215004, China; 5 Department of Urology, The Second Affiliated Hospital, Soochow University, Suzhou 215004, China; 6 Department of Medicine, University of Alabama at Birmingham, Birmingham, AL 35294, USA

**Keywords:** LKB1, survivin, centromere, genome stability

## Abstract

Inactivating mutations in the liver kinase B1 (LKB1) tumor suppressor gene underlie Peutz-Jeghers syndrome (PJS) and occur frequently in various human cancers. We previously showed that LKB1 regulates centrosome duplication via PLK1. Here, we report that LKB1 further helps to maintain genomic stability through negative regulation of survivin, a member of the chromosomal passenger complex (CPC) that mediates CPC targeting to the centromere. We found that loss of LKB1 led to accumulation of misaligned and lagging chromosomes at metaphase and anaphase and increased the appearance of multi- and micro-nucleated cells. Ectopic LKB1 expression reduced these features and improved mitotic fidelity in LKB1-deficient cells. Through pharmacological and genetic manipulations, we showed that LKB1-mediated repression of survivin is independent of AMPK, but requires p53. Consistent with the key influence of LKB1 on survivin expression, immunohistochemical analysis indicated that survivin is highly expressed in intestinal polyps from a PJS patient. Lastly, we reaffirm a potential therapeutic avenue to treat LKB1-mutated tumors by demonstrating the increased sensitivity to survivin inhibitors of LKB1-deficient cells.

## INTRODUCTION

Liver kinase B1 (LKB1) is a serine threonine kinase originally identified as a susceptibility gene for Peutz-Jeghers syndrome (PJS), an inherited disorder characterized by formation of hamartomatous polyps within the gastrointestinal tract [1, 2]. LKB1 functions as a tumor suppressor involved in multiple cellular functions including cell homeostasis, embryogenesis, cell polarity, energy metabolism, cell cycle, and apoptosis. LKB1 is frequently mutated or deleted in several human cancers, and its deficiency in PJS patients also increases cancer risk [3–6]. Although considerable progress has been made in unveiling the diverse functions of LKB1, knowledge of the major biological pathways responsible for its tumor suppressive actions remains incomplete.

Genome-wide screening for mitotic regulators identified LKB1 and its major substrate, adenosine 5‘-monophosphate-activated protein kinase (AMPK), as protein kinases of interest and showed that downregulation of both enzymes induced spindle aberrations in dividing cells [7]. This finding was then further characterized by Wei et al., who showed that loss of LKB1 altered spindle orientation in an AMPK-dependent manner, leading to defective mitosis [8]. Correspondingly, Banko et al. discovered that inactivation of AMPK induces pleiotropic defects in cell mitosis and increases S phase arrest [9]. AMPK-induced phosphorylation is necessary for the function of protein phosphatase-1 regulatory subunit 12C (PPP1R12C), which binds to myosin regulatory light chain and 14-3-3 to dephosphorylate mitotic proteins for mitotic progression and exit [9, 10]. Moreover, activation of AMPKα is required for the association of AMPK with the centrosome, spindle poles, and mid-body during mitosis [8–10]. We recently reported that LKB1 suppresses polo-like kinase 1 (PLK1) and inhibits centrosome over-duplication. In turn, LKB1 loss leads to persistent activation of PLK1, resulting in centrosome amplification and genomic instability [11]. These findings suggest that LKB1 is involved in the regulation of mitosis through both AMPK-dependent and independent mechanisms.

Centromere dynamics also play a crucial role in the maintenance of chromosome integrity during cell division. Defective centromere function results in chromosome segregation errors, which contribute to genomic instability in proliferative diseases like cancer [12–13]. Thus, we asked whether LKB1 may also act on the centromere to regulate genomic stability. By inducing LKB1 expression and analyzing LKB1-deficient cells, we highlight a novel mechanism by which LKB1 preserves centromere integrity during mitosis and prevents abnormal chromosome segregation in mammalian cells. By unmasking a regulatory role of LKB1 on survivin expression, we add further evidence to the potential of survivin inhibitor therapies to target LKB1-deficient tumors.

## RESULTS

### Loss of LKB1 leads to accumulation of misaligned and lagging chromosomes at metaphase and anaphase

**Figure 1:**
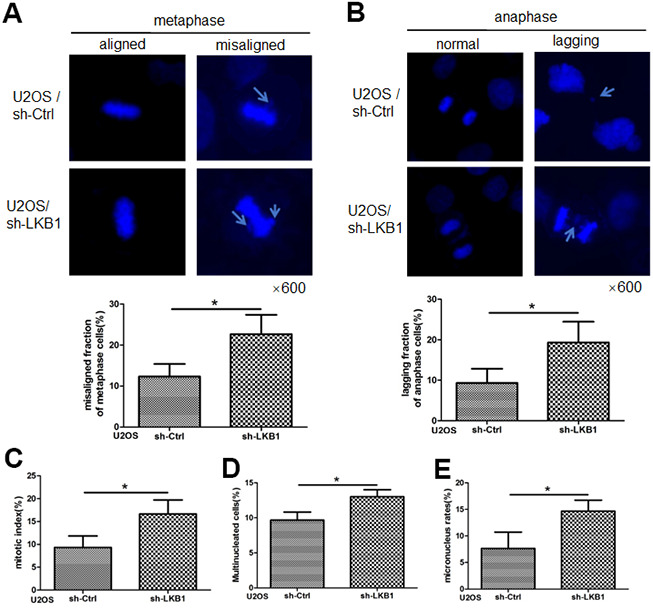
**LKB1 maintains genome stability in U2OS cells.** (**A**) DAPI staining showing accumulation of misaligned chromosomes at metaphase in U2OS/sh-Ctrl and U2OS/sh-LKB1 cells and corresponding data quantification. (**B**) DAPI staining showing accumulation of lagging chromosomes at anaphase in U2OS/sh-Ctrl and U2OS/sh-LKB1 cells and corresponding data quantification. (**C**) Quantification of mitotic index based on p-H3 (Ser10) staining. (**D**) Quantification of multinucleated cells. Cells with 2 or more nuclei were counted. (**E**) Quantification of micronucleated cells. Chromosome segregation defects were recorded in 100 randomly selected mitoses from each experiment (n=3). Data are mean ± SD; *P<0.05.

Chromosome misalignment and lagging are major reasons leading to chromosome mis-segregation and genomic instability. To determine the impact of LKB1 on chromosome stability, we performed shRNA-mediated knockdown of the LKB1 gene (shR-LKB1) in U2OS cells and evaluated the resulting chromosomal changes [14]. As shown in Figure 1A, a few misaligned chromosomes were observed in control cells at metaphase. In contrast, an increase of almost 100% in the frequency of the misaligned chromosomes was detected in cells transfected with shR-LKB1. Lagging chromosomes, resulting from separation or orientation defects during migration of sister chromatids toward opposing spindle poles during anaphase/telophase, were also observed in the latter cells (Figure 1B). In addition, downregulation of LKB1 led to an increase in the mitotic index (Figure 1C), reflecting compromised checkpoint function, accumulation of multinucleated (Figure 1D), and micronucleated (Figure 1E) cells.

**Figure 2:**
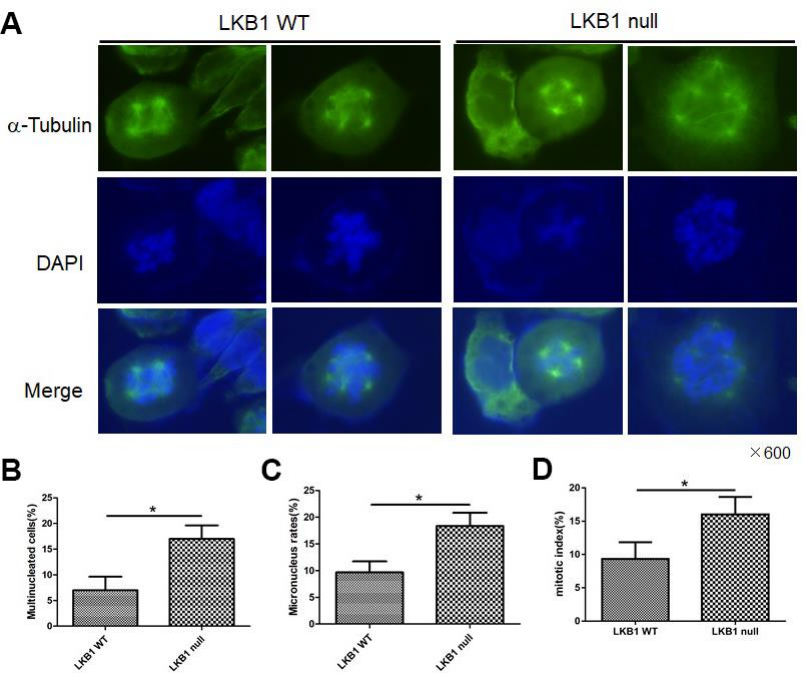
**LKB1 deletion leads to genome instability.** (**A**) Alpha-tubulin staining of proliferating LKB1-WT and LKB1-null cells. (**B**) DAPI staining showing increased multinucleation in LKB1-null cells. (**C**) DAPI staining showing increased micronucleation in LKB1-null cells. (**D**) Quantification of mitotic index in LKB1-WT and LKB1-null cells.

To further validate the outcome of LKB1 depletion on cell division and chromosome instability, we examined wild-type (WT) and LKB1-null mouse embryonic fibroblasts (MEFs). Results showed that LKB1 deficiency led to extensive mitotic abnormalities (Figure 2A), a substantial increase in both multinucleated and micronucleated cells (Figure 2B and 2C), and increased the mitotic index (Figure 2D). Thus, we conclude that LKB1 deficiency promotes accumulation of misaligned and lagging chromosomes at metaphase and anaphase, leading to aberrant mitosis and chromosome instability.

### Ectopic LKB1 expression reduces chromosomal aberrations and maintains genome integrity

**Figure 3:**
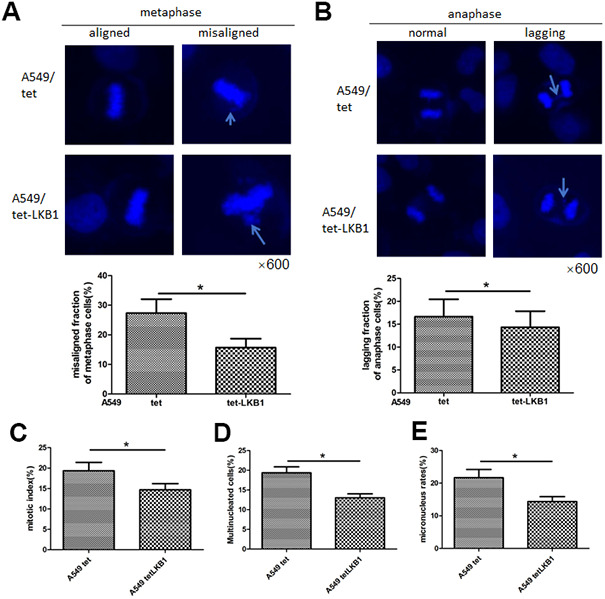
**LKB1 improves genome stability in LKB1-deficient A549 cells.** (**A**) DAPI staining showing accumulation of misaligned chromosomes at metaphase in A549 cells and corresponding data quantification. (**B**) DAPI staining showing accumulation of lagging chromosomes at anaphase in A549 cells and corresponding data quantification. (**C**) Mitotic index quantification based on p-H3 (Ser10) staining. (**D**) Quantification of multinucleated cells. (**E**) Quantification of micronucleated cells. Chromosome segregation defects were recorded in 100 randomly selected mitoses from each experiment (n=3). Data are mean ± SD; *P<0.05.

To further validate the impact of LKB1 on chromosome integrity, we inducibly expressed LKB1 in A549 cells (i.e. A549/tet-LKB1), a lung adenocarcinoma cell line lacking endogenous LKB1. Ectopic expression of LKB1 reduced the occurrence of misaligned and lagging chromosomes (Figure 3A, 3B), activated the mitotic checkpoint (Figure 3C), and reduced the number of multinucleated and micronucleated cells (Figure 3D, 3E).

### LKB1 suppresses survivin expression

**Figure 4:**
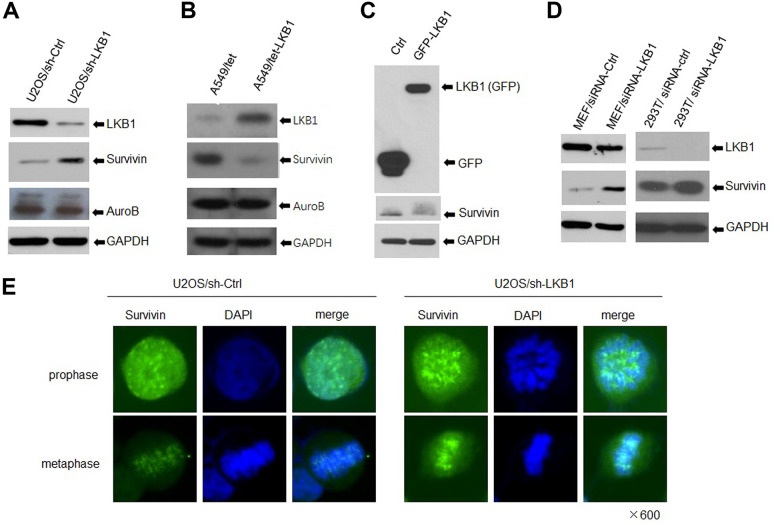
**LKB1 represses survivin expression.** (**A**) Western blotting detection of survivin and Aurora B in U2OS/sh-Ctrl and U2OS/sh-LKB1 cells. (**B**) Western blotting detection of survivin and Aurora B in A549/tet (control) and A549/tet-LKB1 cells. (**C**) Western blotting detection of survivin in Hela cells after induced expression of LKB1. (**D**) Western blotting detection of survivin in MEF, 293T, and C33A cells after LKB1 knockdown. Thirty micrograms of protein per lane were loaded. GAPDH served as loading control. (**E**) Immunofluorescence staining showing centromeric localization of survivin in U2OS cells.

The chromosomal passenger complex (CPC), formed by Aurora B, inner centromere protein (INCENP), survivin, and Borealin, helps maintain centromere integrity to assure proper chromosome segregation during mitosis [12, 14]. To determine whether LKB1 regulates chromosome stability by impacting the centromeres, we detected the expression of Aurora B and survivin by western blotting. Results showed that survivin expression was markedly increased, whereas Aurora B expression was slightly decreased, in LKB1 knockdown cells (Figure 4A). In contrast, in A549 and HeLa cells with inducible expression of LKB1, survivin levels were decreased, whereas the expression of Aurora B was not altered noticeably (Figure 4B, 4C). Furthermore, after LKB1 knockdown, a similar survivin expression pattern was observed in MEFs, 293T, and C33A cells (Figure 4D). Consistent with previous reports, immunofluorescence assays showed that survivin was condensed into small, highly intense foci at the centromeres in prophase and metaphase in U2OS cells (Figure 4E), and its fluorescence was markedly increased after LKB1 knockdown (i,e, U2OS/sh-LKB1 cells) (Figure 4E).

**Figure 5:**
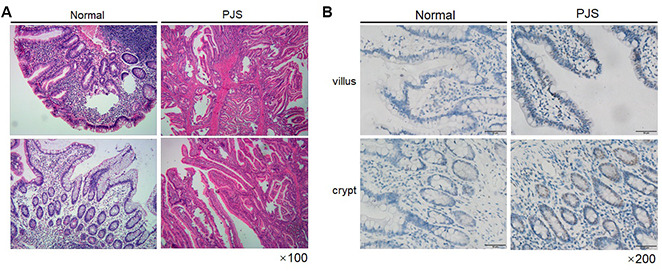
**Survivin expression is increased in intestinal PJS polyps.** (**A**) HE staining of an intestinal polyp from a PJS patient. (**B**) IHC detection of survivin expression in intestinal villi and crypts from PJS and control samples.

To further validate the effect of LKB1 on survivin expression, we analyzed intestinal tissue from a PJS patient with LKB1 deletion confirmed by pathology (data not shown). Hematoxylin-eosin (HE) staining of the PJS sample revealed a typical polypoid phenotype (Figure 5A). Importantly, we confirmed through IHC that the expression of survivin was much higher in the intestinal villi and crypts of the PJS sample, compared with intestinal tissue from an individual without the disease (Figure 5B). Taken together, our results demonstrated that expression of survivin is inversely correlated with that of LKB1.

### Survivin mediates chromosomal aberrations and genome instability induced by LKB1 deficiency

**Figure 6:**
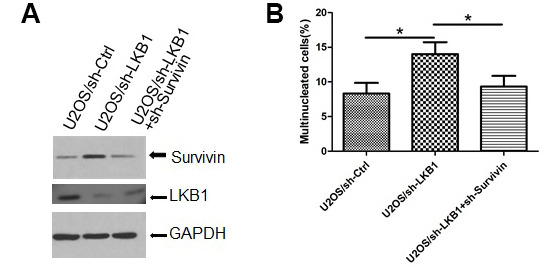
**Survivin mediates chromosomal and genomic instability induced by LKB1 deficiency.** (**A**) Western blotting detection of survivin expression after transfection of sh-survivin into U2OS/sh-LKB1 cells. (**B**) DAPI staining showing multinucleated cells after sh-survivin transfection.

To gain insight into the role of survivin in the mitotic defects induced by LKB1 depletion, we examined nuclear and chromosomal changes in U2OS/sh-LKB1 cells in which survivin was silenced via shRNA. Results showed that transfection of sh-survivin substantially reduced the accumulation of misaligned and lagging chromosomes at metaphase and anaphase, and decreased also the number of multinucleated cells (Figure 6A, 6B, and data not shown).

### LKB1 inhibits survivin expression independently of AMPK

To determine whether LKB1 decreases survivin levels through transcriptional or post-transcriptional mechanisms, we measured the stability of survivin after LKB1 knockdown in U2OS cells treated with cycloheximide (CHX), a protein synthesis inhibitor. Western blot analyses at different time points showed no differences in survivin half-life between control and LKB1 knockdown cells (Supplementary Figure 1).

AMPK is a main downstream effector of LKB1 [15–16]. To determine whether LKB1 affects the expression of survivin through AMPK, we first assessed survivin expression in WT and AMPK-null MEFs. Western blot results showed AMPK deficiency appreciably reduced survivin levels (Supplementary Figure 2A). Nocodazole, a reagent that induces cell cycle arrest in G2/M phase, substantially activated AMPK in WT MEFs as indicated by increased phosphorylation of its substrate acetyl-CoA carboxylase (ACC), a rate-limiting enzyme in mitochondrial fatty acid β-oxidation. However, nocodazole induced the accumulation of survivin in both WT and AMPK-null MEFs, indicating both AMPK-dependent and independent regulation of survivin expression (Supplementary Figure 2A). To further assess the role of AMPK activation on survivin expression, we transfected AMPK-CA (constitutively active) or AMPK-DN (dominant negative) constructs into U2OS cells. As shown in Supplementary Figure 2B, survivin levels increased after LKB1 knockdown regardless of AMPK status. Thus, we conclude that LKB1 regulates survivin expression incompletely dependent on AMPK.

### LKB1 inhibits survivin transcription via p53

**Figure 7:**
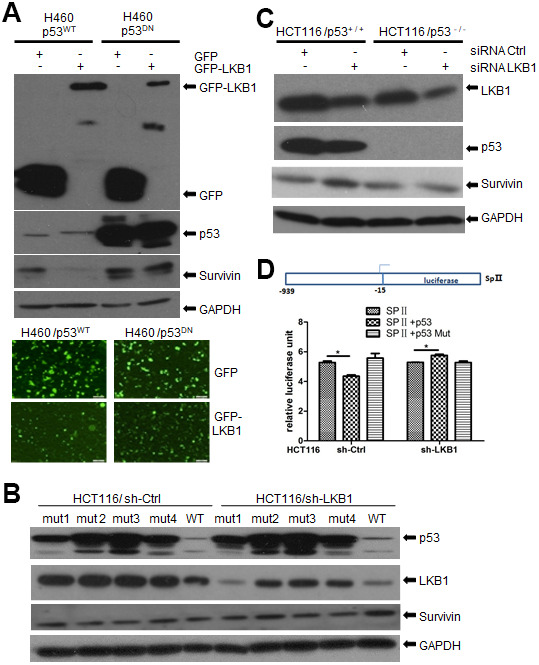
**LKB1 suppresses survivin expression via p53.** (**A**) Western blotting detection of survivin in p53WT and p53DN H460 cells after overexpression of LKB1. (**B**) Western blotting detection of survivin in HCT116/sh-Ctrl and HCT116/sh-LKB1 cells after transfection with mutated P53 (#1-#4) or WT P53 vectors. (**C**) Western blotting detection of survivin in HCT116(p53^-^/^-^) and HCT116(p53^+^/^+^) cells transfected with siRNA-Ctrl or siRNA-LKB1. Thirty micrograms of protein per lane were loaded. GAPDH served as loading control. (**D**) Luciferase reporter assay indicating negative regulation of the survivin promoter by p53.

Previous reports showed that activation of p53 represses the transcription of survivin and reduces survivin protein levels in multiple cancer cell lines. However, recent studies demonstrated that survivin may also affect chromosome and genome stability independently of p53 [17, 18]. Since LKB1 was reported to bind p53 [19], we asked whether LKB1 could suppress survivin through interaction with p53. To answer this question, we examined survivin expression after ectopic LKB1 expression in p53WT and p53DN H460 cells (which contain a mutation that renders LKB1 inactive). Consistent with previous reports, survivin levels were lower in cells ectopically expressing WT p53, compared with p53DN-transfected cells (Figure 7A). In turn, ectopic expression of LKB1 decreased survivin levels only in p53WT cells, but not in p53DN cells (Figure 7A). Next, we transfected mutated- and WT-p53 into HCT116 cells lacking endogenous p53. Expression of WTp53 inhibited survivin expression in both HCT116/sh-Ctrl and HCT116/sh-LKB1 cells (Figure 7B). In contrast, survivin expression remained relatively high in p53-mutant cells regardless of LKB1 status. Using western blot, we also examined survivin levels in HCT116 p53(^+^/^+^) and HCT116 p53(^-^/^-^) stable cell lines before and after LKB1 knockdown. Basal survivin expression was lower in p53(^+^/^+^) cells than in p53(^-^/^-^) cells (Figure 7C). Following LKB1 knockdown, survivin expression was increased in p53(^+^/^+^) cells, but only marginally affected in p53(^-^/^-^) cells. This suggests that LKB1 negatively regulates survivin expression by interacting with p53.

To further examine the suppressive effect of p53 on survivin transcription, the survivin promoter containing the binding sequence for p53 was cloned upstream of the firefly luciferase reporter gene to create the reporter construct SpII (Figure 7D) [20]. The SpII construct was then introduced along with WT or mutant p53 expression vectors into p53-null HCT116 cells in which LKB1 was knocked down (sh-LKB1) or left intact (sh-Ctrl). As shown in Figure 7D, WTp53 repressed survivin promoter activity in HCT116/sh-Ctrl cells, whereas mutant p53 had no effect. In contrast, WT53 did not reduce promoter activity in HCT116/sh-LKB1 cells. These results support the conclusion that LKB1-induced suppression of survivin is dependent on p53.

### LKB1-deficient cells are sensitive to survivin inhibitors

**Figure 8:**
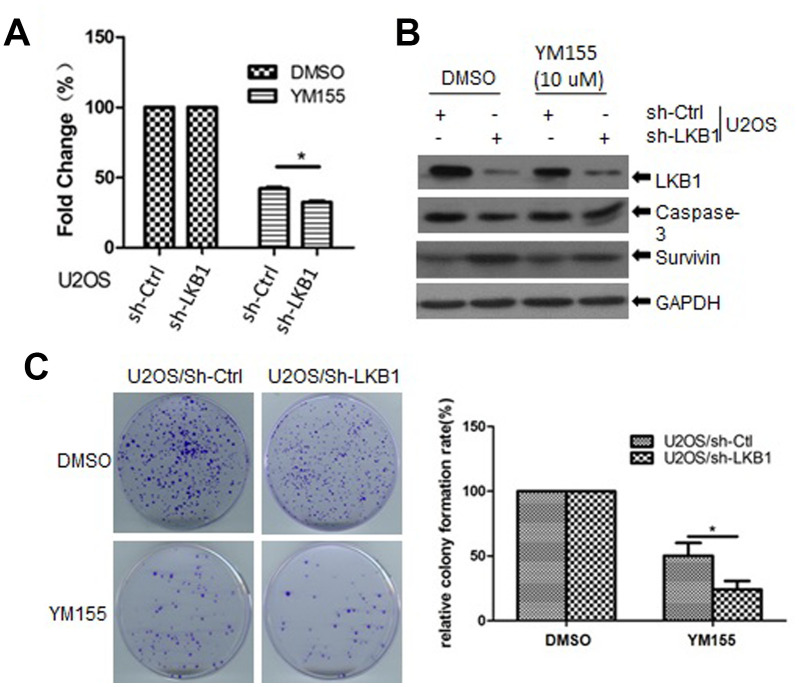
**LKB1 deficiency sensitizes cells to the survivin inhibitor YM155.** (**A**) Determination of cell viability (MTT assay) in U2OS/sh-Ctrl and U2OS/sh-LKB1 cells treated for 24 h with 10 μM YM155. (**B**) Western blotting detection of caspase-3 expression in U2OS/sh-Ctrl and U2OS/sh-LKB1 cells treated with YM155 (24 h, 10 μM). (**C**) Assessment of colony-forming efficiency in U2OS/sh-Ctrl and U2OS/sh-LKB1 cells. Cells were treated with 10 μM YM155 keeping or vehicle and allowed to grow for 10-14 days. Colonies containing at least 50 cells were counted. Data are mean ± SD (n=3).

To determine the impact of LKB1 deficiency on cellular sensitivity to survivin inhibition, we examined cell viability in control and LKB1 knockdown U2OS cells treated with YM155, a survivin inhibitor. We found that cell viability was reduced, while apoptosis was increased, in LKB1 knockdown cells exposed to YM155 (Figure 8A, 8B). In addition, colony-forming assays showed that after YM155 treatment, colony survival reached, respectively, ~22% and ~50% in LKB1-deficient and LKB1-competent U2OS cells (Figure 8C). Thus, we conclude that LKB1 depletion or inactivation increases cell sensitivity to survivin inhibition.

## DISCUSSION

Liver kinase B1 (LKB1), also known as serine/threonine protein kinase 11 (STK11), was first identified as the causative gene in inherited Peutz–Jeghers syndrome (PJS). Subsequent studies reported that LKB1 gene mutations also occur in sporadic cancers, including gastric, colorectal, lung, breast, and cervical cancers. LKB1 functions as a tumor suppressor that becomes activated upon binding to STE20-related adapter (STRAD) pseudokinase and mouse protein-25 (MO25), a scaffolding protein. Upon translocation of this heterotrimeric complex to the cytoplasm, LKB1 activates 13 members of the AMPK family of kinases to regulate a wide range of cellular functions [21–23]. Reports indicated that LKB1 bears important functions in the nucleus as well. Kline et al. reported that decreased nuclear LKB1 correlates with metastatic spreading of head and neck squamous cell carcinoma (HNSCC), emphasizing a nuclear function of LKB1 in repressing HNSCC metastasis [24]. LKB1 can be phosphorylated by several DNA damage sensors, including DNA-dependent protein kinase (DNA-PK), ataxia-telangiectasia mutated (ATM) and ATM- and Rad3-Related protein (ATR), and its deficiency impairs the DNA damage response [25–28]. Acting in the nucleus, LKB1 also plays a key role in mitotic regulation [10, 11]. In a previous study, we reported that loss of LKB1 leads to persistent activation of PLK1, hence promoting centrosome amplification and genomic instability [11].

Centromere dynamics are also critical in the maintenance of genome stability [12], but whether they are influenced by LKB1 remains unknown. In this study, we examined the impact of LKB1 knockdown and ectopic expression and characterized a novel mechanism by which LKB1 exerts control over centromere stability and chromosome segregation**.** LKB1 knockdown in UOS2 cells and MEFs led to misaligned chromosomes at metaphase and lagging chromosomes at anaphase, abnormal chromosomal segregation, and promoted formation of micronucleated and multinucleated cells. Conversely, ectopic expression of LKB1 reduced the occurrence of misaligned and lagging chromosomes and decreased multinucleation and micronucleation in A549 cells, which lack a functional LKB1 protein. Analysis of Aurora B and survivin, which contribute to centromeric stability as part of the CPC [12, 29], showed that survivin expression was markedly increased after LKB1 knockdown in UOS2 cells, and reduced instead after induced expression of LKB1 in A549 cells. Supporting these in vitro findings, we demonstrated that survivin expression was elevated in intestinal polyps of a PJS patient, compared to normal intestine specimens.

Initially characterized in its role as an apoptosis inhibitor [30–31], survivin acts also during early mitosis when it associates around centromeres with Aurora B, Borealin, and INCENP to form the CPC, responsible for sensing and correcting non-bipolar microtubule-kinetochore interactions and ensuring proper segregation of chromosomes [32–34]. Tight regulation of survivin levels at the centromere is essential during prophase and metaphase, and mitotic disorders ensue if its expression is out of range [33–34]. Accordingly, we showed here that survivin knockdown partially reversed mitotic defects and multinucleation induced by LKB1 silencing in U2OS cells.

Using CHX to inhibit protein synthesis, we showed that relative survivin expression levels remained constant regardless of LKB1 status in U2OS cells. Therefore, we investigated the transcriptional mechanisms involved in LKB1-mediated repression of survivin. To this end, we first focused on AMPK, a major downstream target of LKB1. Interestingly, we found that survivin levels were decreased in AMPK-null MEFs but remained unchanged after nocodazole-mediated activation of AMPK in both AMPK-null and AMPK-competent cells. Moreover, LKB1 silencing reduced survivin expression even after induced expression of a constitutively active form of AMPK. Therefore, these data strongly indicate that LKB1 downregulates survivin independently of AMPK. Research showed that LKB1 is a mediator of p53-dependent apoptosis [35] and that p53 is in turn a potent transcriptional activator of the LKB1 gene [36]. Moreover, since LKB1 was shown to be required for the transcription of p21/WAF1 and other p53-regulated genes [19], we analyzed LKB1-dependent survivin expression in HCT116 p53(^+^/^+^) and HCT116 p53(^-^/^-^) cells. These experiments showed that p53 was indeed essential for suppression of survivin expression by LKB1. Lastly, considering that LKB1 is frequently mutated or deleted in various cancers, we analyzed the response of LKB1-deficient cells to YM155, a small imidazolium-based compound that blocks survivin expression via transcriptional inhibition of the survivin gene promoter [37–39]. We found that LKB1 depletion increased sensitivity to YM155 in U2OS cells, as reflected by a reduction and an increase, respectively, in the proliferation and apoptosis rate. These results may provide further stimulus in examining LKB1 status to identify cancer patients who might be responsive to survivin inhibitor drugs.

In summary, we conclude that LKB1 inactivation leads to centromere defects and genome instability via de-repression of survivin expression through a mechanism likely involving direct interaction between LKB1 and p53. Of note, these results are consistent with the recent reporting of an inverse relationship between LKB1 and survivin expression in patients with gastric carcinoma, as well as significant associations between LKB1, p53, and survivin expression levels and overall survival rate [40].

## MATERIALS AND METHODS

### Cell culture

The human U2OS, H460, HCT116, and wild-type and LKB1-null MEFs were maintained in Dulbecco's Modified Eagle Media (DMEM) supplemented with 10% fetal bovine serum (FBS, Atlanta Biologicals, Inc., Flowery Branch, GA, USA) at 37 °C in 5% CO_2_. C33A (cervical cancer) cells were maintained in MEM with 10% FBS, 1% non-essential amino acids, and 1% sodium pyruvate. HeLa cells expressing GFP-LKB1 were stored in our lab and maintained according to a previous report [11].

### Antibodies

Antibodies against phospho-AMPKa (T172) (#2535) and pan-AMPKa (#2532) were purchased from Cell Signaling Technologies (Boston, MA, USA). Antibodies against GFP (clone B-2, #sc-9996), LKB1 (clone ley37D/G6, #sc-32245), and survivin (clone D-8, #sc-17779) were purchased from Santa Cruz Biotechnology (Santa Cruz, CA, USA). GAPDH antibody (#A300-640A) was obtained from Bethyl Laboratories (Montgomery, TX, USA). Polyclonal (#SAB3501072) and monoclonal (clone B-5-1-2, #T5168) antibodies against α-tubulin were purchased from Sigma-Aldrich (St. Louis, MO, USA).

### siRNA and over-expression vector transfection

We followed siRNA methods described in our previous report [11, 13]. A pool of four siRNA duplexes targeting human or mouse LKB1 and a non-targeting siRNA pool were purchased from Dharmacon, Inc. (Lafayette, CO, USA). The siRNA sequences were reported previously [13]. over-expression Cells were grown in a six-well plate to 40% confluence and then incubated with a mixture of a 60 nmol siRNA duplex and 5 μl solution of DharmaFECT 1 Transfection Reagent (Dharmacon, Inc.). After 4 h, FBS was added to a final concentration of 10% (v/v). For over-expression, transfected cells were treated with 100 μM zinc to induce protein expression [11].

### Stable LKB1 knockdown

Five premade lentiviral LKB1 short hairpin RNA (shRNA) constructs and a negative control construct were purchased from Open Biosystems (Huntsville, AL, USA). Lentiviral helper plasmids (pCMV-dR8.2 dvpr and pCMV-VSV-G) were obtained from Addgene (Cambridge, MA, USA). Lentivirus stocks were prepared according to the manufacturer’s protocol. To select for U2OS and C33A cells stably expressing shRNA constructs, cells were plated at sub-confluent densities and infected with 1 mL of virus-containing medium with 10 μg/mL polybrene. Selection with 0.5 to 1 μg/mL of puromycin was started at 48 h after infection. After 2 weeks of selection, surviving cells were harvested for use and cryopreservation.

### Immunofluorescent staining

Cells grown on coverslips were fixed with 4% paraformaldehyde at room temperature for 15 min, permeabilized with 0.25% Triton X-100 in PBS for 10 min, and blocked with 1% bovine serum albumin (BSA) for 20 min. Next, the coverslips were incubated with primary antibodies for 1 h at room temperature, washed with PBS, and incubated with secondary antibodies for 1 h. After a final wash with PBS, the coverslips were mounted using anti-fading mounting medium containing 4, 6-diamidino-2-phenylindole (DAPI). Fluorescent signals were observed with an Olympus IX53 microscope.

### Western blotting analysis

Cells were lysed using RIPA buffer (50 mM Tris–HCl pH 7.5, 150 mM NaCl, 1% Nonidet P-40, 0.5% sodium deoxycholate, 0.1% sodium dodecyl sulfate). Equal amounts of protein were separated using 6–15% SDS–PAGE, followed by electrotransfer onto a polyvinylidene difluoride membrane (Thermo Scientific, USA). The membranes were blocked with 5% nonfat milk for 1 h and incubated with primary antibodies at room temperature. After exposure to corresponding horseradish peroxidase (HRP)-labeled secondary antibodies, the membranes were developed using an enhanced chemiluminescence detection system (GE Healthcare, USA).

### Colony formation assay

U2OS cells (1 × 10^3^) were plated in medium, treated with vehicle or YM155 keeping, and grown for an additional 10-14 days. The resulting colonies were fixed with cold methanol and stained with 0. 5% crystal violet. Colonies containing ≥50 cells were counted to estimate the fraction of surviving cells, calculated as the ratio of plating efficiency between treated and untreated cells. Means and standard deviations were computed from three independent experiments.

### Survivin luciferase reporter assay

The survivin promoter (-938 to +15 bp), ligated to the promoterless firefly luciferase gene (pGL2-basic, Promega) to create the SpII construct, was a gift from Dr. Maureen Murphy of Fox Chase Cancer Center, Philadelphia [20]. Proliferating HCT116 cells were transfected with 4 μg of SpII, along with WT or mutant p53 expression plasmids, using CalFectin™. Fifty ng pRL vector expressing wild-type Renilla luciferase was used as a control reporter. After 48 h, the cells were harvested and lysed, and dual-luciferase assays were performed as per the protocol from the manufacturer (Promega) on a Monolight 2010 luminometer (Analytical Luminescence Laboratory). The ratio of Sp luciferase activity to Renilla activity (relative luciferase unit, RLU) was calculated to estimate survivin promoter activity.

### Immunohistochemistry

Paraffin-embedded intestinal tissue sections were dried at 70°C in an oven for 1 h, dewaxed three times in xylene, hydrated in graded concentrations of ethanol, and washed twice in water. Sections were then treated with ethylenediaminetetraacetic acid (1 mM) for antigen retrieval, washed three times (5 min/wash) in PBS with 0.05% Tween 20 (PBST), and blocked in 5% BSA for 1 h. After incubation with survivin antibody at 4°C overnight, sections were washed in PBST, incubated with HRP-labeled secondary antibodies for 30 min, washed in PBST, stained with diaminobenzidine (DAB), and counterstained with hematoxylin. Finally, the sections were dehydrated in graded concentrations of ethanol, mounted on slides, and observed under a microscope.

### Statistical analysis

Data were analyzed by SAS 9.3 software and are presented as mean ± standard deviation (SD). Differences between groups were analyzed using Student’s *t* test. P<0.05 was considered significant.

## Supplementary Material

Supplementary Figures
