# Supplementary material for: LKB1 inactivation leads to centromere defects and genome instability via p53-dependent upregulation of survivin
Source: Aging (Albany NY). 2020 Jul 16;12(14):14341–54. doi: 10.18632/aging.103473 (PMC7425461; doi:10.18632/aging.103473)
Supplement: Supplementary Figures [file aging-12-103473-s001..pdf]

SUPPLEMENTARY FIGURES

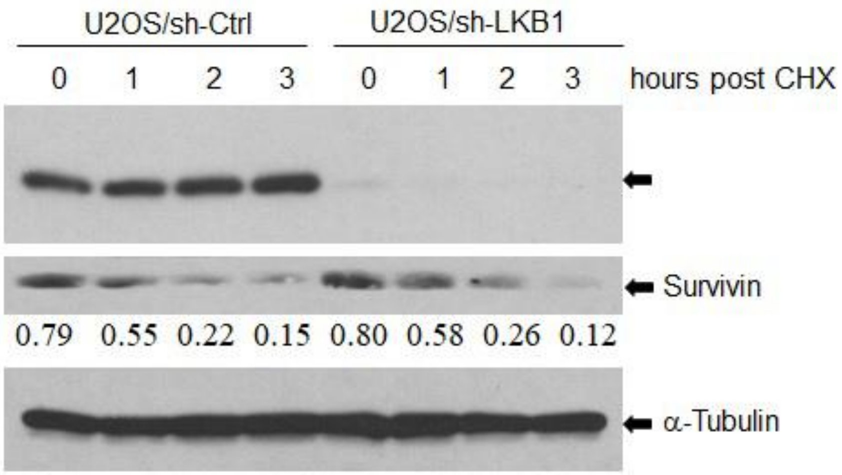

Supplementary Figure 1. Western blotting results showing that LKB1 does not influence survivin protein stability after CHX addition (20 µg/mL).

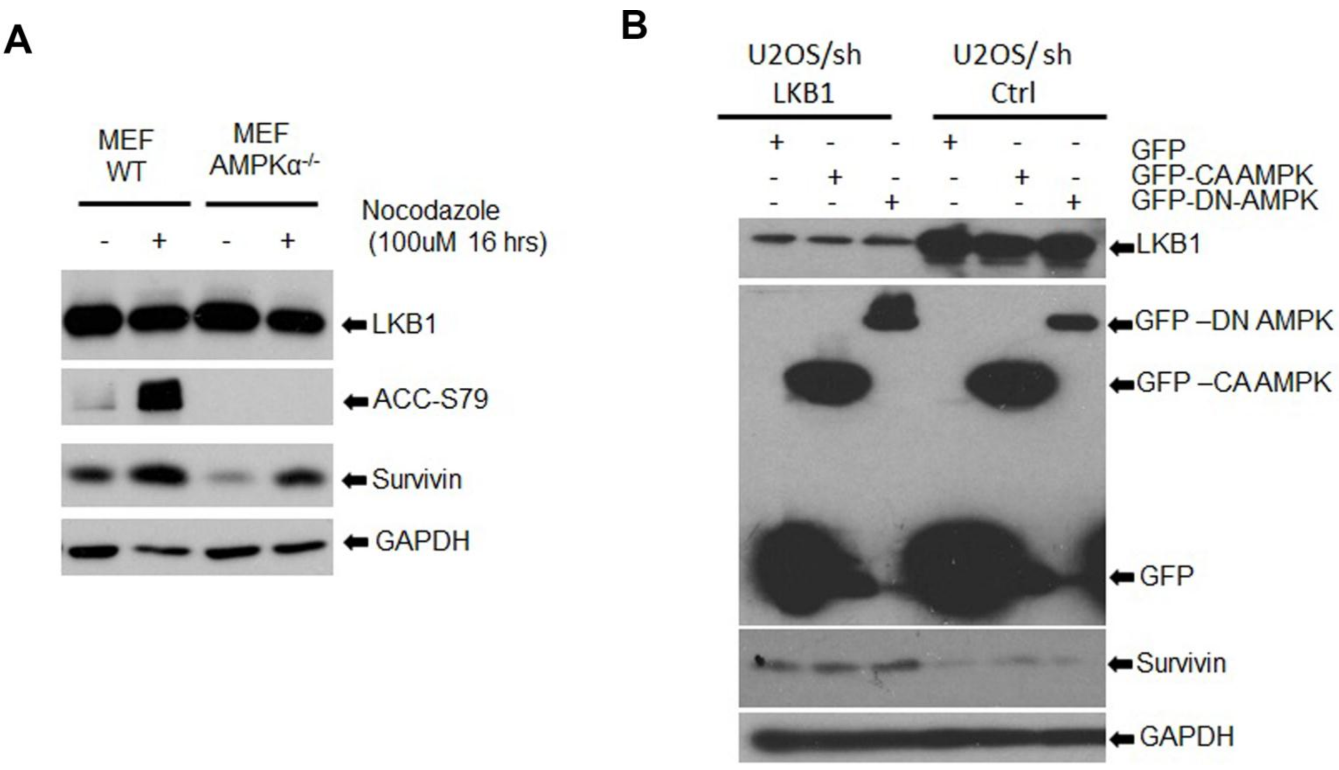

Supplementary Figure 2. LKB1 suppresses survivin expression independently of AMPK. (**A**) Survivin expression was detected using western blotting in WT and AMPKα$^{-/-}$ MEFs cells exposed to nocodazole or control vehicle. (**B**) Transfection of GFP-DN-AMPK and GFP-KA-AMPK into U2OS cells and survivin detection by western blotting. Thirty micrograms of protein per lane were loaded. GAPDH served as loading control.
